# Acute undifferentiated leukemia with undifferentiated myeloid sarcoma: Case report and literature review

**DOI:** 10.1097/MD.0000000000036948

**Published:** 2024-01-26

**Authors:** Lan Luo, Xiaoqing Wang, Ji Luo, Shuai Zheng, Ninghan Gong, Yuan He, Qian Xi, Jiao Chen, Tao Jiang, Ling Zhong

**Affiliations:** a College of Medical Technology, Chengdu University of Traditional Chinese Medicine, Chengdu, Sichuan, China; b Department of Hematology, Sichuan Academy of Medical Sciences and Sichuan Provincial People’s Hospital, School of medicine, University of Electronic Science and Technology of China, Chengdu, Sichuan Province, China; c University of Electronic Science and Technology of China, Chengdu, Sichuan, China; d Department of Clinical and Experimental Medicine, Sichuan Academy of Medical Sciences and Sichuan Provincial People’s Hospital, School of medicine, University of Electronic Science and Technology of China, Chengdu, Sichuan Province, China; e Department of pathology, Sichuan Academy of Medical Sciences and Sichuan Provincial People’s Hospital, School of medicine, University of Electronic Science and Technology of China, Chengdu, Sichuan Province, China; f Human Disease Genes Key Laboratory of Sichuan Province, Sichuan Provincial People’s Hospital, University of Electronic Science and Technology of China, Chengdu, China.

**Keywords:** acute undifferentiated leukemia, case report, myeloid sarcoma

## Abstract

**Background:**

With the advancement of diagnostic technology, true acute undifferentiated leukemia (AUL) is becoming more rare, and AUL with extramedullary sarcoma has not been reported.

**Case presentation:**

This article reports a case of AUL with extramedullary sarcoma. Flow cytometric analysis of the bone marrow and lymph nodes indicated that the tumor cells of both were of the same origin and mainly expressed stem cell markers and CD7, no myeloid-specific markers, T-lymphoblastic-related markers, and B-lymphoblastic-related markers. Although the priming regimen combined with azacitidine was ineffective, complete remission was achieved by switching to azacitidine combined with HIA (homoharringtonine, idarubicin plus Ara-C).

**Conclusion:**

To diagnosis de novo acute leukemia with extensive and comprehensive cellular immune maker detection is available and credible, the expression of a single relatively nonspecific myeloid antigen as a immune maker to detect AUL or AUL associated with sarcoma is precise and effective in our case, which patient was benefit from HIA regiment.

**The novelty statements**

To the best of our knowledge this is the first reported case of acute undifferentiated leukemia (AUL) with undifferentiated myeloid sarcoma and successfully treated by HIA regimen. We found to diagnose AUL extensive and comprehensive cellular immune maker detection is mandatory and extramedullary involvement was best detected by flow cytometry (FCM).

## 1. Introduction

AUL is a rare type of acute leukemia characterized by the absence of lineage-specific differentiation antigens and loss of myeloid antigen expression, with a low incidence rate and poor prognosis.^[1]^ Myeloid sarcoma, a special type of myeloid tumor that manifests as soft tissue masses rather than bone marrow involvement, can occur alone or accompanied by acute myeloid leukemia (AML). The most commonly involved sites are the orbits and lymph nodes, also called chloroma. There are few reports of undifferentiated myeloid sarcoma, and because specific antigens are rarely expressed, it is easily confused with histiocyte tumors. AUL is rare owing to its clinical features, primarily bone marrow involvement, and cases accompanied by extramedullary sarcoma have not been reported. Here, we describe a case of AUL with undifferentiated myeloid sarcoma and present a review of the literature.

## 2. Case report

**Figure 1.**
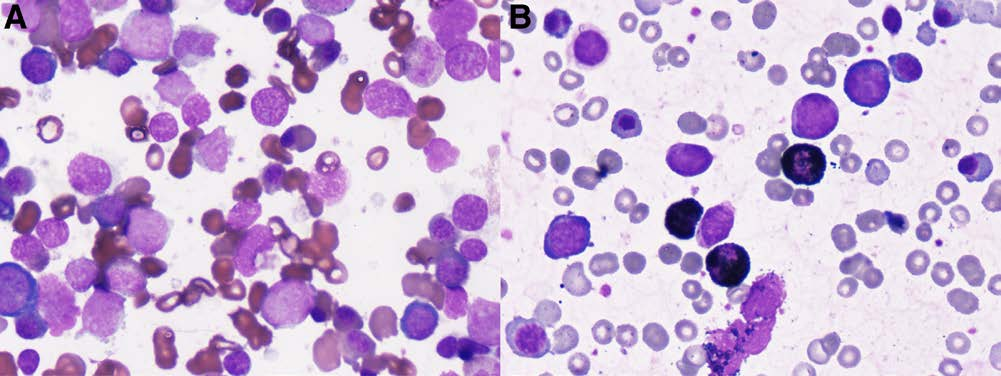
Blasts in the bone marrow. (A) Wright staining showed a significant increase in blasts. (B) Peroxidase staining of blast cells was negative.

**Figure 2.**
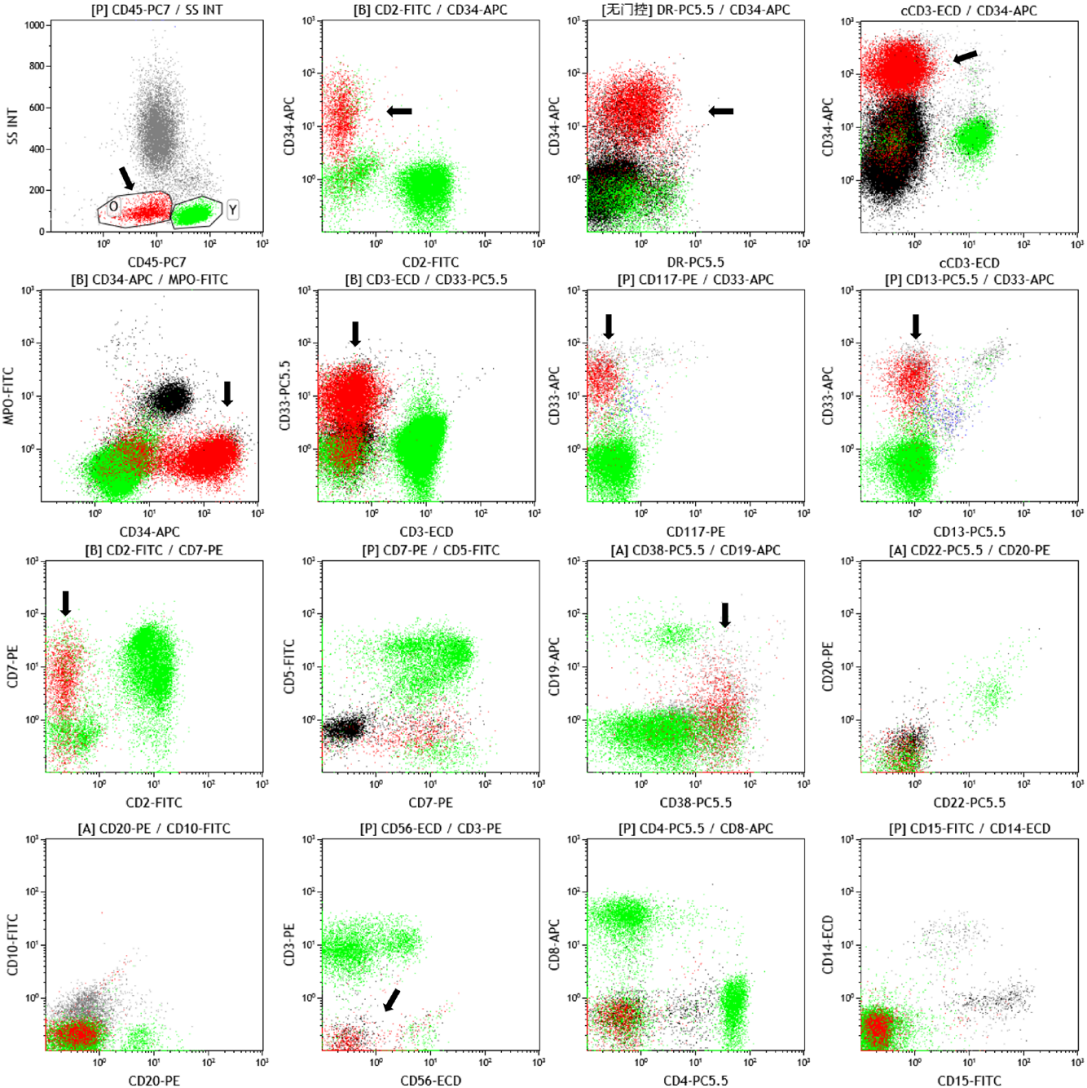
Bone marrow aspirate immunophenotypic analysis by flow cytometry confirmed an abnormal cell population with 28.23% nucleated cells. The cell population was positive for CD34, CD33 and CD38; some cells expressed CD7 and HLA-DR, and a few expressed CD56. CD2, CD19, CD20, CD10, CD13, CD117, CD15, CD14, MPO, cCD79a and cCD3 were not expressed.

**Figure 3.**
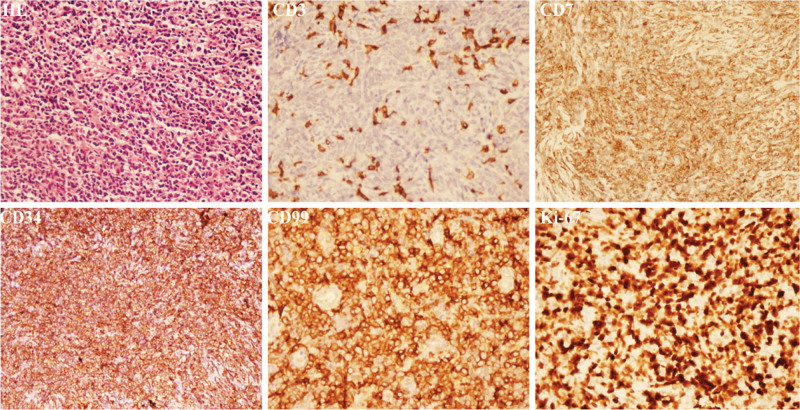
Lymph node biopsy showed that the structure of the lymph node was destroyed (HE). Immature cells expressed CD34, CD99, and CD7 but did not express CD3. Ki-67 staining revealed active tumor proliferation.

A 23-year-old male patient visited our hospital on October 11, 2020. His chief complaint was cough for 4 months with fever. Antibiotic treatment was ineffective. His medical history was unremarkable. Physical examination showed signs of anemia; the bilateral submandibular and left axillary lymph nodes were palpable, the right submandibular lymph node was 1.5 cm × 1.5 cm, and the left submandibular lymph node was 4 cm × 4 cm. No organomegaly was observed. CBC revealed a white blood cell count of 3.670 × 10^9^/L, neutrophil count of 1.402 × 10^9^/L, hemoglobin 44 g/L, platelet count of 437 × 10^9^/L, and 7.5% immature cells. Biochemistry tests showed LDH 559 U/L, and coagulation function tests were normal. Based on bone marrow aspiration from the left posterior superior iliac spine, proliferation of nucleated cells in the bone marrow was active, with a granulocyte to erythrocyte ratio of 0.50:1. The proportion of blasts was increased, accounting for 16.0% of ANC (Fig. 1). FCM analysis of the bone marrow was performed; 50,000 cells were obtained and analyzed. In a CD45/SSC scatter plot, 28.23% of immature cells with an abnormal immunophenotype were detected in the CD45 dim and SS low area. CD34, CD33 and CD38 were positive. CD7 and HLA-DR were partially positive, and CD56 was dim. CD2, CD19, CD20, CD10, CD13, CD117, CD15, CD14, MPO, cCD79a and cCD3 were negative (Fig. 2). A diagnosis of myelodysplastic syndrome with excess blast 2 was made, and azacytidine 100 mg qd on days 1 to 7 plus a 14-days priming regimen (G-CSF 300 µg qd d2–17, homoharringtonine 1 mg qd d3–17, cytarabine 15 mg bid d3–17) was started. As lymph node enlargement in MDS is rare, we performed another bone marrow test and lymph node biopsy. Bone marrow aspiration from the right posterior superior iliac spine on the 12th day of induction chemotherapy showed that blasts accounted for 24.5% of ANCs, which met the criteria for acute leukemia. FCM was consistent with the previous immunophenotype of bone marrow blasts. Karyotype results were 90 to 91 < 4n>, XXYY, add(2)(q37) × 2, 6, i(17)(q10) × 2[6]/46,XY[14]. A somatic frameshift mutation, c.1236dup (p. Gln413Thrfster13), in the ETV6 gene was detected in the bone marrow sample. FCM of the left cervical lymph node sample showed 36.4% immature cells with an abnormal immunophenotype in CD45 dim/low SS areas expressing CD34, CD33 and CD7 but not CD2, CD10, CD5, CD4, CD8 and CD117. Immunohistochemistry of immature cells in the left cervical lymph node showed the following: CD7 (+), CD43(+), CD3(−), CD2(−), CD5(−), CD4(−), CD8(−), CD10(+), CD34(+), CD99(+), TdT(−), CD20(−), PAX-5(−), CD15(−), MPO(−), CD21(−), CD117(−), CD123(−), PGM-1(−), KP1(−), CK(−), and Ki-67 90% (Fig. 3). The EBER1/2 in situ hybridization result was tumor cells (−). Therefore, a diagnosis of AUL with undifferentiated granulocytic sarcoma was considered. The chemotherapy plan was changed to the HIA regimen (homoharringtonine 3 mg d1-3, idarubicin 10 mg d1-3, cytarabine 1.5 g q12 h d1-3) due to the high blasts in bone marrow after 12 days of chemotherapy, and the patient achieved complete remission after the regimen was changed. After CR, the patient was scheduled for consolidation chemotherapy and stem cell transplantation. Unfortunately, he died of sepsis due to myelosuppression after a scheduled 2^nd^ consolidation chemotherapy cycle.

## 3. Discussion and conclusions

This patient was a rare case of AUL accompanied by lymph node masses. Lymph node flow cytometric analysis showed that the immunophenotype of lymph node tumor cells was consistent with the immunophenotype of leukemia cells from his bone marrow, which was consistent with AUL. According to biopsy, the lymph node structure was destroyed, and immunohistochemistry showed that the tumor cells of the lymph nodes and bone marrow were identical and consistent with AUL. Therefore, this case was in line with a diagnosis of AUL with extramedullary sarcoma. We conducted a literature search, and to the best of our knowledge, this is the first report of AUL with extramedullary sarcoma.

AUL belongs to acute leukemias of ambiguous lineage and is characterized by lack of lineage-specific antigens in leukemia cells and expression of no or only one myeloid leukemia-related immune marker.^[2]^ This type of leukemia is extremely rare. A total of 1888 AUL cases diagnosed from 2000 to 2016 are found in the US SEER database, with an incidence rate of approximately 1.34 persons/million.^[2]^ With the advancement of diagnostic technology, the number of true AUL cases has been decreasing yearly.^[2]^ Patients with AUL are often older, with a median age of 75 years, and 70% of patients are over 60 years old. Compared with other AMLs, AUL has a worse prognosis, and the median survival time is significantly shorter than that of AML.^[3]^ The diagnosis of AUL mainly relies on bone marrow FCM to analyze immune markers of leukemia cells.^[3]^ The WHO defines AUL as a leukemia that expresses neither lymphoid markers nor myeloid markers and for which NK-cell precursors, basophils, and even nonhematopoietic tumors need to be excluded. Weinberg et al reported 24 cases of AUL, with no significant difference from AML-M_0_ in terms of age of onset, blood cell count, degree of bone marrow hyperplasia, and ratio of bone marrow blasts.^[3]^ In terms of immunophenotype, myeloid markers, including CD13, CD33, and CD117, were not expressed in 6 cases; in 15 cases, 1 myeloid marker was partially or completely expressed, and 1 myeloid marker plus another myeloid marker were partially or weakly expressed in 3 cases. In our case, blasts from both the bone marrow and lymph nodes expressed only one myeloid marker, CD33. In Weinberg study, B cell markers such as CD19, CD20, and CD10 were not expressed in 24 cases of AUL, but in 5 cases, cytosolic CD22 or cytosolic CD79a was partially expressed.^[3]^ No case expressed cCD3 or sCD3, but expression of other T cell-related antigens was common, with CD7 being the most common. In AUL, blasts rarely express more than one monocyte marker; 2 cases expressed CD11b, and only one case was partially positive for nonspecific esterase. The blasts in our patient expressed CD7 but were negative for sCD3 and cCD3, and no monocyte marker was positive.

AML with minimal differentiation, also known as AML-M_0_, is a more common AML subtype with no morphological or cytochemical evidence of myeloid differentiation and can easily be confused with AUL. Myeloid maturation of blasts in AML-M_0_ is demonstrated by immunological markers. The most vital myeloid marker, MPO, is often negative by cytochemistry but may be positive in some blasts by FCM or immunohistochemistry. Blasts of AML-M_0_ express at least 2 myeloid-associated markers, usually CD13, CD117, and CD33. CD7 expression is reported in approximately 40% of AML-M_0_ cases. In 2016, the WHO noted that expression of a single, relatively nonspecific myeloid-associated antigen, especially by only some blasts and along with other markers of primitive cells (e.g., CD7, CD34, and HLA-DR), is more typical of AUL than AML-M_0_. There is still another subtype of leukemia that may be confused with AUL-acute leukemia of ambiguous lineage, not otherwise specified in which combinations of markers that do not allow for their classification as AUL are expressed. In AUL, no more than one membrane marker for any given lineage is typically expressed. Hence, if a given lineage expresses more than one marker, a diagnosis of acute leukemias of ambiguous lineage, not otherwise specified, would be appropriate.

Myeloid sarcoma is a type of myeloid neoplasm in which myeloid tumor cells invade the extramedullary tissues and destroy the original tissue structure to form a mass.^[4]^ It can occur independently of AML, concomitantly with AML, and after bone marrow remission.^[5]^ Myeloid sarcoma is common in AML-M_0_, AML-M_4_ and AML-M_5_. Myeloid sarcoma can affect any site of the body, most often involving the skin, lymph nodes, gastrointestinal tract, bone, soft tissue and testes.^[6]^ Myeloid sarcoma usually expresses myeloid tumor-related markers, though undifferentiated myeloid sarcoma is rarely seen. In 1995, Tosi et al reported a case of epidural undifferentiated granulocytic sarcoma that occurred before acute promyelocytic leukemia, which was cured by treatment with all-trans retinoic acid, but the case was not truly undifferentiated due to a lack of FCM immunophenotyping.^[7]^ In our case, lymph node puncture and FCM and immunohistochemistry were performed. FCM confirmed that tumor cells from the lymph nodes had the same immunophenotype as leukemia cells from the bone marrow, indicating that the tumor cells from the lymph nodes were the same as the leukemia cells from the bone marrow of this patient. Immunohistochemistry results were also consistent with FCM results. Combined with the bone marrow immunophenotype, this case was consistent with AUL, indicating undifferentiated myeloid sarcoma in the affected lymph nodes.

## Author contributions

**Data curation:** Ji Luo, Shuai Zheng, Ninghan Gong.

**Methodology:** Xiaoqing Wang, Yuan He, Qian Xi.

**Writing – original draft:** Lan Luo.

**Writing – review & editing:** Jiao Chen, Tao Jiang, Ling Zhong.
